# 3D Lattices of Core/Shell Ge/Mn Quantum Dots in an Alumina Matrix: Structure, Fabrication, and Photo-Electrical Properties

**DOI:** 10.3390/nano14231906

**Published:** 2024-11-27

**Authors:** Ivana Periša, Gabrijela Svalina, Mile Ivanda, Marija Tkalčević, Sigrid Bernstorff, Maja Mičetić

**Affiliations:** 1 Ruđer Bošković Institute, Bijenička Cesta 54, 10000 Zagreb, Croatia; ivana.perisa@irb.hr (I.P.); gsvalina@irb.hr (G.S.); ivanda@irb.hr (M.I.); 2 Dipartimento di Ingegneria Industriale, University of Salerno, Via Giovanni Paolo II, 132, 84084 Fisciano, Italy; mtkalcevic@unisa.it; 3 Elettra-Sincrotrone, S.C.p.A., Basovizza, 34149 Trieste, Italy; sigrid.bernstorff@elettra.eu

**Keywords:** quantum efficiency, magnetron sputtering, germanium, core/shell quantum dots, multiple exciton generation, manganese

## Abstract

Materials consisting of quantum dots with a semiconductor-core, metal–shell structure often have exciting and tunable photo-electrical properties in a large range of values, and they are adjustable by core and shell structure parameters. Here, we investigated the influence of Mn-shell addition to Ge quantum dots formed in an alumina matrix by magnetron sputtering deposition. We show a well-achieved formation of the 3D regular lattices of Ge-core, Mn-rich shell quantum dots, which were achieved by self-assembled growth mode. Intermixing of Ge and Mn in the shell was observed. The optical, electrical, and photo-conversion properties were strongly affected by the addition of the Mn shell and its thickness. The shell induced changes in the optical gap of the materials and caused an increase in the material’s conductivity. The most significant changes occurred in the photo-electrical properties of the materials. Their quantum efficiency, i.e., the efficiency of the conversion of photon energy to the electrical current, was very strongly enhanced by the shell addition, though it depended on its thickness. The best results were obtained for the thinnest shell added to the Ge core, for which the maximal quantum efficiency was significantly enhanced by more than 100%. The effect was, evidently, the consequence of multiple exciton generation, which was enhanced by the shell addition. The obtained materials offer great potential for various applications in photo-sensitive devices.

## 1. Introduction

The development of new, as well as the improvement of existing, photosensitive devices is currently the focus of numerous researchers [1,2,3]. Since these devices find application in diverse industries, their improvement can significantly contribute to different sectors and enable progress in various technological fields.

Nanostructured semiconductor materials, especially quantum dots (QDs), represent an up-and-coming group of materials that can be used for improving the performance of these devices [4,5,6,7,8,9]. For instance, the utilization of semiconductor QDs can significantly enhance the power conversion efficiency of photovoltaic devices. What enables this improvement are their unique properties [10]. Their properties significantly differ from those of corresponding macro materials and can be fine tuned by manipulating the material size and their spatial arrangement. These specific properties are a consequence of the quantum confinement effect [11,12]. This effect occurs when one or more dimensions of a material become comparable to its Bohr exciton radius, leading to a change in the electronic structure of the material. The semiconductor bandgap increases and the shape of the electronic state density undergoes significant changes [13,14]. In the case of quantum dots, this results in the discretization of states, allowing for the excitation of more than one exciton for a single absorbed photon, which is known as multiple exciton generation (MEG) [15,16]. This increases the quantity of generated photocurrents, which leads to the aforementioned enhancement of the power conversion efficiency. Additionally, the change in the semiconductor bandgap leads to a modification of the optical absorption of these materials. Therefore, altering the material’s dimensionality and controlling the spatial arrangement of quantum dots enable the creation of its various properties (depending on the desired application).

Germanium (Ge) QDs are particularly interesting for the aforementioned purpose [17,18]. An example of this is the successful utilization of Ge QDs for enhancing the quantum efficiency (QE) of a photodetector. Ge is specific because of its large Bohr radius of excitons (24 nm), which offers broad possibilities for manipulating quantum properties. Consequently, in Ge nanostructures, the quantum confinement effect is very strong and occurs on large scales, enabling a simple manipulation of the material properties (such as tuning the effective bandgap over a wide range of values). Additionally, Ge exhibits a high charge carrier mobility, and the production of Ge quantum structures does not require extremely high temperatures [19,20].

In order to apply or implement these nanostructures into specific systems, it is necessary to embed them into a matrix. In our prior research, we studied the preparation of Ge QDs within various dielectric matrices [21]. It has been shown that the type of matrix significantly influences the structure and formation of quantum dots and, thus, their properties [22]. Another very important aspect of the matrix influence is the achievement of a self-assembling growth regime for QDs. It enables the formation of three-dimensional (3D) regularly ordered QD lattices [23,24]. The self-assembled growth is driven by the surface morphology effects in these systems [23]. It is very important because it ensures a narrower size distribution of the formed QDs and controlled separations of the QDs [24], which are important for the electrical/transport properties of the material.

This research showed that the Ge QDs embedded in alumina (Al_2_O_3_) matrices show very strong confinement effects, but, at the same time, there is one noteworthy problem, namely the oxidation of Ge. To avoid the oxidation of Ge, Ge QDs can be incorporated into a core/shell structure [25]. Previous research has shown that core/shell structures, although they represent more complex systems, offer a range of significant advantages [5,26,27,28]. The shell passivates the core surface, acting as a protective layer to prevent oxidation. Additionally, due to the interaction between the core and shell, the carrier lifetime is increased. Furthermore, it has been demonstrated that the shell significantly enhances the material’s absorption properties and enables manipulation of the absorbed wavelength spectrum. There are several other potential impacts of the core on the shell, such as influencing the tension of the germanium crystal lattice, which is extremely significant for the nature of its energy bandgap, etc. [29]. Therefore, the addition of a shell significantly expands the possibilities for manipulating material properties.

Structures with a semiconductor core and a metal shell, at present, attract strong attention due to their specific properties. Recent studies have unveiled remarkable potential for systems with manganese (Mn) QDs [30,31,32,33]. Overall, Mn QDs offer a unique combination of optical, magnetic, and surface properties, making them promising candidates for a wide range of applications in fields such as electronics, photonics, biotechnology, and medicine. Consequently, exploring the integration of these QDs into the semiconductor core/metal shell system promises to be a particularly intriguing endeavor.

Although previous research on Ge-based core/shell quantum dots shows their exceptional properties, only a few such materials have been explored thus far. Therefore, further research is necessary to fully understand the capabilities of such structures and to optimize them for specific applications.

To effectively harness the advantages of nanostructures, they are fabricated as nanostructured thin films [34]. A variety of techniques for their controlled fabrication have been developed, with particular interest in those based on material sputtering. Their appeal lies in their high suitability for applications in nanotechnology as these techniques allow for a good control of the quantum dot properties by their deposition parameters [35,36]. Additionally, magnetron sputtering enables the fabrication of self-assembled quantum structures arranged in regular three-dimensional (3D) lattices [24]. Such an arrangement of quantum structures is crucial as it achieves much better control of their size and spacing, which are extremely important for applications.

Our study showcases the production of thin-film materials comprising Ge-core and Mn-rich shell QDs embedded within an amorphous alumina matrix. In actuality, the shell consists of Ge and Mn mixture. This was accomplished through a self-assembled growth of a Ge/Mn/Al_2_O_3_ multilayer, which is facilitated by magnetron sputtering deposition. The QDs are arranged in three-dimensional (3D) lattices with a body-centered tetragonal (BCT) structure. In this work, we aimed to demonstrate that it is possible to tune the optical and photo-electrical properties of the material by the thickness of the Mn-related shell. We show a notable enhancement of the material’s quantum efficiency after shell addition to the Ge cores. These findings are relevant for future applications in photo-sensitive devices.

## 2. Materials and Methods

### 2.1. Sample Preparation

To produce the inspected samples, the magnetron sputtering deposition system KJLC CMS-18 was used. During the deposition, the base pressure in the chamber was 8 × 10^−6^ Pa. As a working gas argon was used with a partial pressure of around 0.46 Pa in a continuous flow. The samples were prepared as thin films on Si (100) and glass substrates at a temperature of 400 °C.

Ge/Mn core/shell QDs embedded in an amorphous alumina matrix were produced using Ge/Mn/Al_2_O_3_ multilayer deposition. Twenty Ge/Mn/Al_2_O_3_ tri-layers were produced. Deposition of a Ge layer leads to the formation of Ge QDs. Mn layer deposition covers them via the Mn shells, while alumina grows smoothly serving as a matrix [5,26]. The size of the Mn shell was tuned by varying the Mn deposition time. The other parameters were kept constant for each sample. Three samples of core-shell QDs and two control samples (only a Ge core without a Mn shell and only an Al_2_O_3_ matrix) were produced. The samples were labeled according to the shell material (Mn) and the respective shell thickness value. Table 1 lists the sample names and sputtering conditions. The sputtering conditions were opted for to provide the self-assembled growth regime so that a 3D lattice of the QDs could be formed [5,26]. This regime is achieved by a specific choice of deposition parameters, ensuring the best influence of surface morphology on the nucleation positions of Ge QDs. As explained in Refs. [23,24], the surface morphology is a driving mechanism for self-assembling growth.

### 2.2. Sample Characterization

The atomic composition of the materials was performed by time of flight elastic recoil detection analysis (TOF-ERDA) with a 6-MV tandem Van de Graaff accelerator at the Ruđer Bošković Institute (Zagreb, Croatia) using 20 MeV 127I^6+^ ions. In order to determine the structural properties of the films, grazing incidence small-angle X-ray scattering (GISAXS), grazing incidence wide-angle X-ray scattering (GIWAXS), and Raman spectroscopy were used. The GISAXS and GIWAXS patterns were simultaneously measured at the synchrotron Elettra at the Austrian SAXS beamline using 8 keV photons, a 2D 100 K Pilatus (for GIWAXS), and a 2D Pilatus3 1 M (for GISAXS) detector system (Dectris Ltd., Baden, Switzerland). The grazing incidence angle exceeded the critical angle of total reflection by a small margin. The GIWAXS detector was set to measure the intensity in the plane that was vertical to the sample surface. In order to be able to analyze the measured GISAXS maps, we used the paracrystal model. More details about it and some more examples of its use can be found in our previous papers [37,38]. As has been mentioned, Raman spectroscopy was also used. This method was applied to gain an insight into the pure Ge-Ge bonds. A DILOR Z-24 triple monochromator and an argon ion laser with an excitation line of 514.5 nm were used for these measurements. Furthermore, the analysis of the optical properties was performed using Ocean Optics equipment (Orlando, FL, USA), including a deuterium–halogen light source (DH-2000-BAL), a UV/VIS detector (HR4000), and SpectraSuite software, version 2.0. Finally, the electrical properties, such as quantum efficiency and electrical resistance, were inspected. They were carried out using a PTS-2-QE System from Sciencetech (London, ON, Canada) covering the spectral range from 320 nm to 1200 nm. For this purpose, two contacts were deposited via magnetron sputtering deposition, one over the film (a transparent indium-doped tin oxide) and the other one on the bottom of the substrate (aluminum). The quantum efficiency was measured using a bias voltage of 5 V, while the resistance was measured in the dark using a bias voltage of 3 V.

## 3. Results and Discussion

### 3.1. Structural Properties

#### 3.1.1. Atomic Composition

The elemental composition of the prepared films was determined by TOF-ERDA measurements. These measurements were crucial for verifying the quantity of the Ge and Mn present within the QDs. The findings of the analysis are presented in Table 2. The Ge and Mn atomic percentages (Ge at% and Mn at%) and the total number of the corresponding atoms (Ge 10^15^ at/cm^2^ and Mn 10^15^ at/cm^2^) are given, as well as the total number of the all atoms present in the films (film thickness 10^15^ at/cm^2^). The number of Ge atoms was nearly constant in the films with Mn, while it was slightly lower in the film without Mn. This difference usually occurred due to the slightly changed deposition conditions when one additional sputtering target (Mn in this case) was active. The Mn concentration was observed as increasing with longer deposition time, which was consistent with the deposition conditions. Any slight fluctuations in the Mn concentration were encompassed within the statistical error.

#### 3.1.2. Geometrical Properties

The GISAXS method was used to investigate the core/shell structure of the QDs and their ordering properties. This technique is a highly effective method for core/shell structure investigation since it is susceptible to very tiny sizes and shells that are merely a few atoms thick. This method enables the determination of the QDs’ shape, size, ordering properties, and their statistical distribution [37,39].

**Figure 1:**
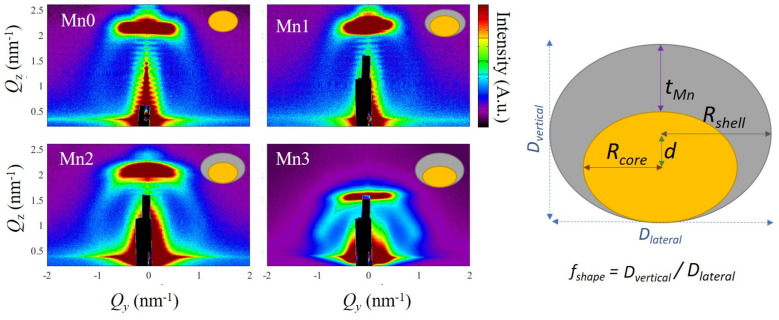
GISAXS maps of all the prepared thin films. The insets illustrate schematic representations of the various QD structures found in the inspected films, with the Ge core depicted in yellow and the Mn-related shell in gray. The QD parameters (*R*_core_, *R*_shell_, *t*_Mn_, *d*, and *f*_shape_) used in the analysis and the GISAXS maps are shown in the schematic illustration of QD.

Figure 1 presents the GISAXS maps of all the investigated films. All of the investigated maps displayed a peak arrangement that is typical of a 3D lattice formation of QDs with a BCT arrangement [21]. In other words, all of the GISAXS maps showed horizontal sheets, implying the existence of a multilayer structure within the films, which was centered at *Q*_y_ = 0 nm^−1^. Additionally, the prominent peaks (Bragg spots) were visible at *Q*_z_~1 nm^−1^, confirming the existence of a 3D regular arrangement of Ge QDs.

The GISAXS maps were numerically analyzed using the paracrystal model [37], which were embedded in the GisaxStudio software, version 060 [38] to obtain the structural parameters. This model assumes that the QDs are organized within a 3D BCT lattice and that the QDs have a core/shell internal structure. The formed BCT lattice is characterized by the basis vectors ***a***_1_ to ***a***_3_; |***a***_1_| = |***a***_2_| = *a* is the separation of QDs in the plane parallel to the substrate; and |***a***_3z_| = *c* is multilayer period. The QDs are assumed to have spheroidal shape characterized by core radius *R*_core_, shell radius *R_s_*_hell_, and shape factor *f*_shape_, which is equal to the ratio of the vertical (perpendicular to the films surface) and lateral (parallel to the films surface) radius (*R*_core_ and *R_s_*_hell_) or the corresponding diameters (*D*_vertical_ and *D*_lateral_). The scheme of the formed QDs and their main parameters are given in Figure 1. Another important feature is that the center of the core was shifted to with respect to the center of the shell for the amount *d*, so the core was, in actuality, placed at the bottom of the shell *d = f*_shape_ (*R*_shell_ − *R*_core_), as visible in Figure 1. The shell thickness is denoted as *t*_Mn_, and it was, in actuality, double the value of the shell thickness of the centered (symmetrical) core shell QD. Therefore, the shell exhibits asymmetry, resembling a cap enveloping the upper portion of a QD. A detailed explanation of the model can be found in Ref. [37].

Due to the small amount of Mn atoms in the materials, and the large number of the fitting parameters, the constraint on the size of the formed QDs was set for the fitting process. The initial QD size, i.e., the values of the core and shell radii were calculated from the atomic percentages of Ge and Mn and the geometrical parameters of the formed BCT QD lattice (*a* and *c*). The parameter *c* is very precisely determined from the position of horizontal Bragg sheets, while the parameter *a* can be well estimated from the position of the lateral Bragg peaks. The density of the amorphous Ge and crystalline Mn were taken for the calculation. These values were taken as starting parameters for the fitting process. The parameters obtained by the fitting are given in Table 3.

From the obtained results, it follows that the lateral separation (*a*) of the formed QDs increased with the addition of the Mn shell, as well as the multilayer period *c*. The lattice disorder parameters were similar, as expected. The size of the Ge core *R*_core_ slightly increased with the thickness of the Mn shell. The increase was expected because the lateral separation of the QDs increased, while the number of Ge atoms was nearly constant, so the formed QDs should have been larger for the larger lateral separation.

The thickness of the Mn-related shell (*t*_Mn_) increased, aligning with the intended deposition parameters. However, the obtained thicknesses were larger than expected, if the formation of pure Mn shells were assumed. This was calculated using the same procedure of atomic composition estimation from the geometrical parameters of the formed QD lattice and the sizes of the core and shell. In actuality, it was possible to obtain a good agreement between the GISAXS parameters and the measured atomic composition only if a mixed Ge + Mn shell was assumed. The calculated atomic percentages of the materials from the GISAXS parameters assuming the formation of a Mn_x_Ge_1−x_ shell with *x* = 0.5 are given in the last two columns of Table 3. The values obtained agree well with the results of the TOF-ERDA measurements from Table 2. The precise determination of *x* was not possible due to relatively large errors in the determination of the GISAXS parameters. Additionally, the shell thicknesses were quite small, so there were many interface atoms that were possibly bonded to the matrix.

The formed QDs were slightly flattened (*f*_shape_~0.6–0.8) and the values of the radii standard deviations were about 0.3 nm.

Generally, the GISAXS method is very sensitive to small structural changes, including core–shell structure of QDs; therefore, it is very suitable for structural analysis. In Ref. [40] comparative GISAXS was shown, as was the transmission electron microscopy (TEM) analysis of very similar system. Ge/Si core/shell QDs were also arranged in a BCT lattice. It was shown there that GISAXS is much more sensitive then TEM in the analysis of these very small QDs with a core/shell structure. However, the initial parameters for the fit, or the setting of constraints on some parameters, are very important.

#### 3.1.3. Crystalline Properties

**Figure 2:**
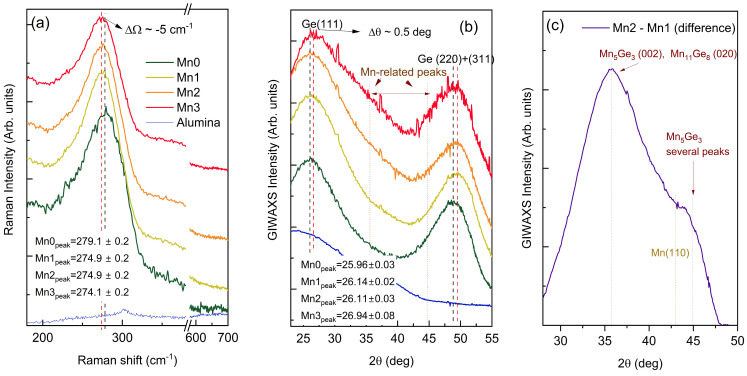
(**a**) The Raman spectra of the prepared thin films. The positions of the peaks for the film without a shell (Mn0) and with the largest shell (Mn3) are indicated by the black and red dashed lines, respectively. (**b**) The grazing incidence wide-angle X-ray scattering (GIWAXS) of the same films. The black dashed line indicates the positions of the Ge (111) and Ge (220 + 311) peaks corresponding to the core material (MN0). The red dashed line marks the position of the same peaks for the film with the largest shell (Mn3). The positions of the peaks are indicated in the figure. (**c**) The difference of the GIWAXS intensity of the Mn2 and Mn1 films, which show similar positions to the Ge-related peaks. The observed difference was attributed to the intermixing of Ge and Mn at the core/shell interface.

To determine the crystalline structure of the prepared materials, GIWAXS and Raman measurements were conducted. Figure 2 outlines the results obtained.

The notable peaks observed in both measurement types showed the presence of either amorphous Ge or very small Ge crystallites, while the alumina matrix was also fully amorphous, which is consistent with the findings from previous studies on similar systems [21]. The Raman spectroscopy data (Figure 2a) revealed a significant Ge-related band that was close to 275 cm^−1^, and it is typical for amorphous Ge. No peaks corresponding to Mn oxides were visible (they appear near 650 cm^−1^), suggesting the formation of a Mn or Mn-Ge shell. Intermixing of Ge and Mn should not cause significant change in the Raman spectra according to Ref. [41]. The GIWAXS measurements, as shown in Figure 2b, were in good agreement with the Raman results. The analysis revealed two broad peaks at around 27° and 50°, and these were attributed to the Ge (111) and Ge (200) + Ge (311) peaks. They are characteristic to amorphous Ge or to very small (below 1 nm) Ge crystallites. The GIWAXS spectra of the samples with thicker Mn shells (Mn2 and Mn3) show an increased intensity for 2θ = 30–55 deg. This is the region where the Mn- and Ge-Mn-related peaks appear [42]. This indicates a formation of some Ge-Mn binary alloys at the Ge/Mn interface. To better analyze this effect, the difference of the GIWAXS intensity of the Mn2 and Mn1 films was determined, as shown in Figure 2c. These two films were chosen because they have very similar positions to the Ge-related peaks and the same Ge-core size. Other films showed a much stronger shift in the peaks and had different Ge core sizes, so they were not suitable for this type of comparison. The strongest contribution was near 36 deg, which may be related to the Mn_5_Ge_3_ and Mn_11_Ge_8_ phases [43]. An additional peak was visible near 45 deg, which can also be related to several Mn_5_Ge_3_ peaks [43]. The peaks were very broad and were consistent with the formation of very thin shells, as found by the GISAXS and TOF-ERDA analyses. The pure Mn peak, Mn (110), should appear at 43 deg, and it is possible that some of the contributions of it existed in the Mn3 film. The observed intermixing of Ge and Mn at the core/shell boundary was found to be in accordance with the GISAXS analysis, which showed good agreement with the elemental composition only if the intermixing of Ge and Mn was assumed. It also can be expected due to the previous investigations of Ge/Mn thin films, which were deposited by magnetron sputtering deposition [43].

Both type of measurements, Raman and GIWAXS, showed shifting in the peaks, and they were dependent on the Mn-rich shell thickness. A shift of the Ge Raman peak was observed with a maximal value of about ΔΩ = −4 cm^−1^ for the Mn3 film with the largest Mn-related shell. This indicates the presence of a biaxial tensile strain in the Ge bonding, which was caused by the addition of a Mn shell [44]. A shift of the GIWAXS peaks was also visible, with a maximal value of Δθ = 0.5 deg for the Mn3 film. In actuality, all of the films with the Mn shell displayed a shift of the peaks. The Raman peaks shifted to smaller angles, whereas the peaks in the GIWAXS measurements shifted to higher values. The shift in the GIWAXS indicated a reduction in the out-of-plane lattice constant, meaning there was an increase in the in-plane lattice constant that was visible in the Raman spectra, which was as a result of biaxial tensile strain [44,45]. The largest peak shift for both types of measurements was observed in the Mn3 sample, which had the largest Mn-related shell. Thus, the peak shift clearly indicated the presence of tensile strain. The in-plane (ε_in_) and out of plane (ε_out_) strain values calculated for different shells are given in Table 4. The observed tensile strain value was obtained using the procedure from Ref. [44]. More precisely, the shift of the Raman peak was ΔΩ = −*b*ε_in_, where *b* = 415 cm^−1^, and ε_in_ is the in-plane strain. The strain from GIWAXS was given by ε_out_ = Δ2θ/(4 × tan(θ)), where 2θ is the position of Ge (111) diffraction peak. The maximal lateral strain of 1.2% was found for the film with the largest Mn-related shell. This is consistent with our previous research [29]. The peak shift, in this case, was significantly smaller compared to our earlier study where the Si_3_N_4_ shell was used to cover Ge QDs. The values of the vertical strain were smaller than the lateral, except for the Mn3 film, where a larger strain was calculated. This can be the consequence of a very strong contribution of the Ge-Mn phase to the GIWAXS intensity, which caused an additional shift of the Ge (111) peak position.

Although the shift of the peak is visible in both types of measurements, we must be aware of the fact that the formed QDs are amorphous and quite small, which can additionally influence the positions and shifts of the peaks. However, according to the deposition conditions, the Ge QDs always form in the same way, and, according to the GISAXS analysis, the sizes of the Ge-cores were quite similar. The second issue was the possible origin of the observed strain. The shell consisted of a Ge-Mn mixture and can also be the origin of the observed shifts. Namely, it was shown that the Ge-Mn alloy has a larger lattice that is constant than pure Ge [46]. Therefore, the larger lattice constant in the shell, which is asymmetric and like a thin cap on the Ge core (see the scheme in Figure 1), can easily produce an increase in the lattice constant in the core in the direction parallel to films surface and, consequently, to its decrease in the vertical direction. But the same effect can be produced by the shell themselves. The shell thickness was very small, even less than single atomic layer for the thinnest shell, and we believe that the origin of the shifts in the Raman and GIWAXS analyses were both parts of the QDs, i.e., the core and the shell.

The strain significantly altered the band structure and optoelectronic characteristics of the semiconductor epitaxial layers. A similar strain was observed in the Ge-core Al-shell QDs investigated in Ref. [29].

### 3.2. Optical Properties

**Figure 3:**
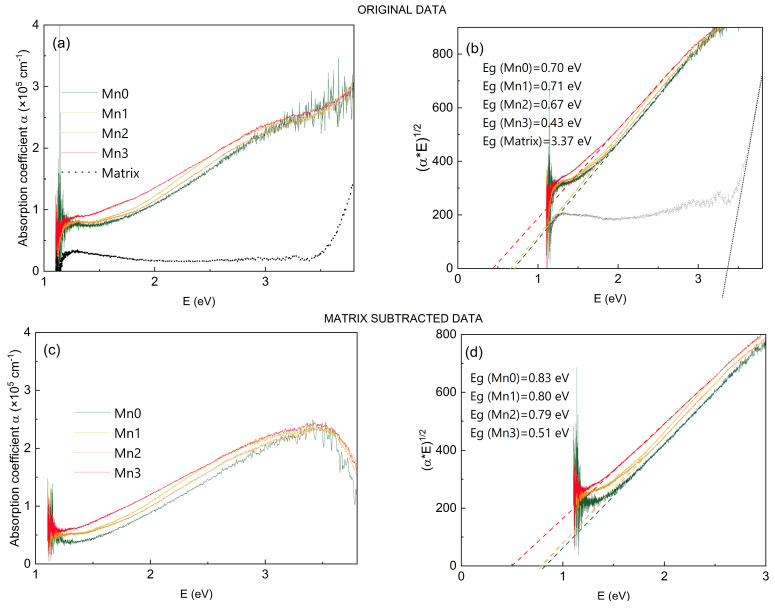
(**a**) The absorption coefficient of the prepared films as a function of the photon energy. (**b**) Optical gap determination using the as-measured optical spectra. The bandgaps values of the thin films were calculated using Tauc’s (αE)^1/2^ plot vs. energy. (**c**) The absorption coefficient of the prepared thin films with a reduced matrix contribution. (**d**) Optical gap determination with a reduced matrix contribution.

The optical properties of the prepared thin films are shown in Figure 3. A slightly higher absorption coefficient was evident for the materials with a Mn shell. These findings indicate that incorporating metallic atoms could boost the photon absorption in QDs, consequently enhancing solar energy utilization. The alumina matrix also showed some lower absorption in the investigated range, as visible in Figure 3a.

The measured spectra were used to determine the optical gap of the materials using the Tauc method [47]. The results are shown in Figure 3b, together with the corresponding Tauc’s plots. Due to the relatively high contribution of the matrix to the total absorption, we also performed the data analysis with the correction to the matrix. The procedure is described in detail in Ref. [48]. The corrected absorption coefficients are given in Figure 3c, while the Tauc gap analysis is given in Figure 3d. For this case, the bandgap gradually decreased with the thickness of the Mn shell. Such behavior is in agreement with our previous investigations of the materials with a Ge core covered with a metallic shell, as described in Ref. [5]. The bandgap of the pure Ge QDs was close to the value of amorphous Ge, while the shell addition caused the bandgap to decrease, which is consistent with the formation of Mn-Ge alloys [49]. According to the results from Ref. [49], the larger Mn_5_Ge_3_ clusters should be metallic, while the smaller and amorphous ones should be semiconductive with a bandgap of 0.45 eV. This value was very close to the bandgap of the Mn3 film, which had the largest Mn shell.

**Figure 4:**
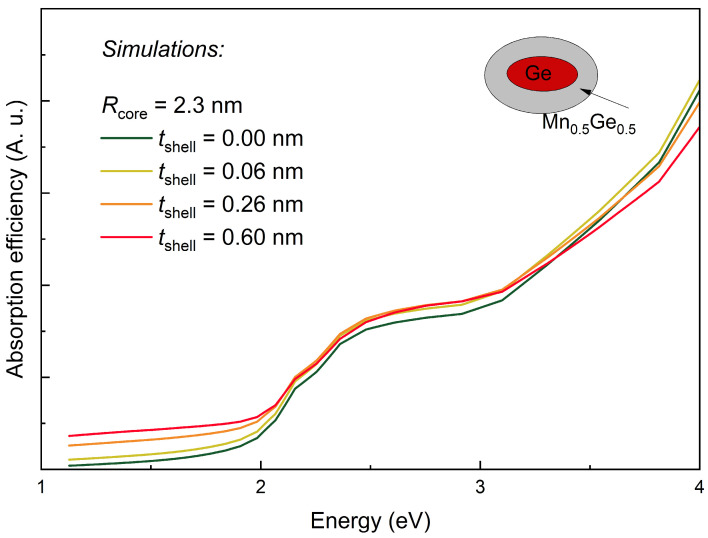
Simulation of the absorption efficiency for the Ge QDs surrounded by a concentric Mn + Ge mixture shell (according to Refs. [50,51]).

We simulated the absorption efficiency using the formulas given in Refs. [50,51] for the spheroidal Ge-core, Mn_0.5_Ge_0.5_-shell QDs that were in an alumina matrix. We used similar values for the radii of the core and shell as were measured for the investigated materials. The outcomes of the simulation are displayed in Figure 4. The simulated curves showed a gradual increase in the intensity and were in good agreement with the experimental data, as shown in Figure 3a. We could not expect an exact matching of the simulations with the experimental results because the used model assumed a concentric core/shell model, as is visible in the inset of Figure 4. Meanwhile, in the experimental case, the core was displaced from the center of the shell, and it was able to induce a slightly different response (Figure 3b–d).

### 3.3. Photo-Electrical Properties

**Figure 5:**
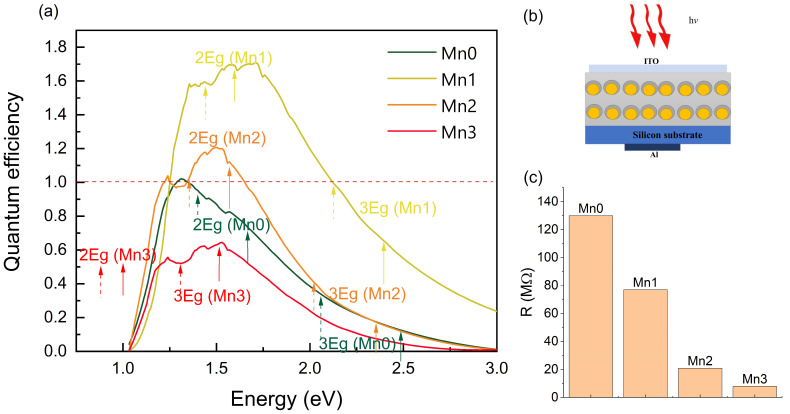
(**a**) The quantum efficiency (QE) of the prepared thin films. The red dashed line represents a QE value of 1, signifying that one exciton is generated for one incident photon. The vertical arrows indicate the position of the calculated bandgap values for each film, which is given in Figure 3d. The color of the arrows relates to the QE line color for the respective film. (**b**) A schematic illustration of the PV devices used for the QE measurements. (**c**) The electrical resistance of the devices, which was measured in the dark.

The quantum efficiency (QE), i.e., the ratio of the number of charge carriers generated with the number of photons absorbed by the coated Ge QDs, was experimentally determined. The results are summarized in Figure 5a, while the used setup for the measurements is schematically illustrated in Figure 5b.

The measurements indicate a strong dependency of the QE on both the presence and thickness of the Mn-related layer. Typically, incorporating a metal-containing shell around a Ge core in thin films can significantly influence the QE, but it is contingent upon the precise design and attributes of the core/shell structure.

For us, the addition of a metal-containing shell both enhanced (Mn1 and Mn2 samples) and decreased (Mn3 sample) the QE values. The best efficiency was obtained for the film with the thinnest shell (Mn1 sample). In this case, the QE value was notably larger than 1, suggesting that multiple exciton generation (MEG) takes place, which is one possible explanation. This means that a single photon produces more than one exciton [15]. In this instance, the QE peak was located at approximately 1.6 eV, which was twice the Mn1 bandgap value of the 0.83 eV calculated for the matrix-corrected spectrum (see Figure 3c). This suggests that the observed phenomenon is a result of two excitons being generated from a single photon excitation. Additionally, this film demonstrated significantly higher QE values at energies exceeding 2.5 eV compared to all the other films. We propose that three exciton generation may also be occurring, contributing to the increased QE values at these higher energy levels. The same observation applied to the Mn2 sample, for which the value of QE was slightly above 1. In this instance, the QE peak was also situated at a position that was twice the Ge QE bandgap value. Another possible explanation of the high QE is due to a trapping of the photogenerated holes on the Ge-related defects/traps. This effect is demonstrated and explained in Ref. [52], in which the Ge QDs formed in a SiO_2_ matrix were investigated. The QDs investigated there were also amorphous, as in our case, so both mentioned mechanisms are possible. Additionally, the enhanced photo-current generation for the thinnest shell is in accordance with our previous research published in Ref. [5]. It was shown there that, theoretically, the thinnest Ta shell causes the strongest electric field enhancement within the Ge core. The effects of the type and thickness of the shell added to the Ge core were quite complex and more detailed research with different shell materials should be performed to understand it better.

The QE of the film with the largest Mn shell was the lowest, showing that thinner shells produce better photo-electric generation properties. The possible explanation for this was that, as the thickness of the Mn-related layer increases, the QDs expand in lateral size and possibly contact one another (*R*_shell_ = 3.2 nm, lateral separation *a* = 6.4 nm), resulting in the formation of a 2D system. This reduces confinement effects and leads to a lower QE. The increased number of defects also caused greater carrier recombination, resulting in lower quantum efficiency and a reduced photocurrent. Overall, this property broadened the detection range toward the lower wavelength region, supporting the findings of Ref. [27].

The resistance of the devices illustrated in Figure 5b is shown in Figure 5c. Evidently, the addition of a Mn shell significantly decreased the resistance. This result was expected due to the metallic character of Mn and the decrease in the QD distance enabling a larger probability of a charge hopping (tunneling) from one QD to another.

To summarize, a significant boost in the QE values was observed in the core/shell nanoparticles compared to the simple Ge particles. This validates the generation of additional extractable charge carriers due to the absorbed photons, demonstrating the potential for utilizing the metal shell to greatly enhance the efficiency of semiconductor nanoparticle solar cells or photodetectors. A strong decrease in the resistance related to the Mn shell thickness was also observed.

## 4. Conclusions

In this work, we explored thin films consisting of core/shell Ge/Mn QDs, which were systematically arranged in 3D BCT lattices in an alumina matrix. We examined how the presence and thickness of the Mn atoms in the shell influenced the structural, optical, electrical, and photo-conversion properties of the material. The results showed a significant impact from both the addition and thickness of the Mn-related shell on these properties. A structural analysis confirmed the existence of a multilayer structure and a 3D regular arrangement of the formed QDs. Additionally, it was demonstrated that Mn was present in the films, with concentrations aligning with the desired deposition conditions. More detailed structural analysis showed an intermixing of the Mn and Ge atoms in the shell and the formation of a Ge-Mn alloy. Therefore, the core/shell QDs, in actuality, consisted of an amorphous Ge core and Ge-Mn alloy shell. This study also revealed the presence of a small tensile strain in the Ge QDs due to the Mn-related shell, which probably influences the optoelectronic properties of the prepared films. The presence of the Mn-related shell also causes an increase in the materials’ absorption. Furthermore, the band gap was shown to gradually decrease with increasing Mn shell thickness. The quantum efficiency was found to strongly depend on the thickness of the Mn-related shell. The quantum efficiency values exceeding 100% were measured for the film with the thinnest Mn-related shell. As expected, the electrical resistance of the prepared devices was found to strongly decrease with the increase in the Mn-atoms in the shell. Simulations of the absorption efficiency closely matched the experimental measurements. In summary, the addition of the Mn-related shell significantly modified the properties of the Ge QDs, making them more versatile for a wide range of applications. All of these findings indicate that these materials are highly suitable for use in diverse photosensitive devices.

## Figures and Tables

**Table 1:** Deposition parameters of the produced QD-containing thin films. *t* denotes the deposition time, and *P* denotes the sputtering power.

Sample	*P*_Ge (W)	*t*_Ge (s)	*P*_Mn (W)	*t*_Mn (s)	*P*_Al_2_O_3_ (W)	*t*_Al_2_O_3_ (s)
Mn0	25	40	0	0	140	200
Mn1	25	40	25	5	140	200
Mn2	25	40	25	10	140	200
Mn3	25	40	25	15	140	200

**Table 2:** The atomic percentage and number of Ge and Mn atoms in the materials.

Sample	Ge at%	Mn at%	Ge 10^15^ at/cm^2^	Mn 10^15^ at/cm^2^	Film Thickness 10^15^ at/cm^2^
Mn0	25 ± 1	0.0	103 ± 8	0	410
Mn1	34 ± 2	0.5 ± 0.1	149 ± 10	2.2 ± 0.5	490
Mn2	32 ± 2	3.2 ± 0.4	152 ± 11	16 ± 2	550
Mn3	24 ± 1	8.7 ± 0.6	146 ± 7	50 ± 7	680

**Table 3:** Parameters of the Ge QD lattices determined from the GISAXS analysis: the QD in-layer separation *a* = |***a***_1_| = |***a***_2_|, multilayer period *c* = |***a***_3z_|, deviations of the QD positions from the ideal ones (*σ*_x_, *σ*_y_, and *σ*_z_), core radius (*R*_core_), shell radius (*R*_shell_), shell thickness (*t*_Mn_), the standard deviation of the sizes distribution *σ*_R_, the shift of the core relative to the center of the shell (*d*), and the shape factor *f*_shape_ (ratio of the vertical to lateral QD radius). The units for each parameter are specified in nanometers (nm). The last two columns show the number of Ge and Mn atoms that were calculated from the GISAXS parameters, assuming the mixture of Ge and Mn to be in an equal ratio within the shell.

Sample	*a*	*c*	*σ*_x_	*σ*_y_	*σ*_z_	*R*_core_	*R*_shell_	*t*_Mn_	*σ*_R_	*d*	*f*_shape_	Ge 10^15^ at/cm^2^	Mn 10^15^ at/cm^2^
Mn0	4.6	2.5	1.9	1.6	0.3	2.10	2.10	0.00	0.3	0.0	0.6	101 ± 7	0
Mn1	5.1	2.7	2.0	1.9	0.3	2.33	2.39	0.08	0.3	0.04	0.6	133 ± 5	7 ± 2
Mn2	5.1	2.8	2.0	1.9	0.3	2.34	2.60	0.34	0.3	0.17	0.66	157 ± 5	21 ± 3
Mn3	6.4	3.4	2.1	1.6	0.3	2.36	3.05	1.05	0.3	0.52	0.76	148 ± 5	59 ± 3

**Table 4:** The positions of the Ge-related peaks in the Raman and GIWAXS analyses and the strain parameters in the planes parallel (ε_in_) and perpendicular (ε_out_) to the films surface, as derived from the Raman and GIWAXS results.

Sample	Peak Position: Raman (cm^−1^)	Peak Position: GIWAXS (deg)	ε_in_ (%)	ε_out_ (%)
Mn0	279.1 ± 0.2	25.96 ± 0.03	---	---
Mn1	274.9 ± 0.2	26.14 ± 0.02	−1.01 ± 0.05	0.34 ± 0.06
Mn2	274.9 ± 0.2	26.11 ± 0.03	−1.01 ± 0.05	0.28 ± 0.06
Mn3	274.1 ± 0.2	26.94 ± 0.08	−1.20 ± 0.05	1.8 ± 0.1

## Data Availability

The raw data supporting the conclusions of this article will be made available by the authors on request.
